# Retraction Note: Down-regulation of LINC00472 promotes osteosarcoma tumorigenesis by reducing FOXO1 expressions via miR-300

**DOI:** 10.1186/s12935-024-03268-7

**Published:** 2024-02-10

**Authors:** Jingwei Zhang, Jieyuan Zhang, Dong Zhang, Weifeng Ni, Haijun Xiao, Bizeng Zhao

**Affiliations:** 1 Department of Orthopedics, Shanghai Fengxian District Central Hospital/Southern Medical University Affiliated Fengxian Hospital, No. 6600 Nanfeng Road, Shanghai, 201499 China; 2 Department of Orthopedics, Shanghai Sixth People?s Hospital, No. 600 Yishan Road, Shanghai, 200233 China

**Retraction Note: Cancer Cell International (2020) 20:100**

10.1186/s12935-020-01170-6

The Editors-in-Chief have retracted this article because the authors were not able to provide documentary evidence of approval from an appropriate ethics committee for the work reported. Haijun Xiao agrees with this retraction. The remaining authors did not respond to correspondence from the publisher about this retraction.

